# Snapshot linear-Stokes imaging spectropolarimeter using division-of-focal-plane polarimetry and integral field spectroscopy

**DOI:** 10.1038/srep42115

**Published:** 2017-02-13

**Authors:** Tingkui Mu, Shaun Pacheco, Zeyu Chen, Chunmin Zhang, Rongguang Liang

**Affiliations:** 1 College of Optical Sciences, University of Arizona, Tucson, Arizona 85721, USA; 2 Institute of Space Optics, School of Science, MOE Key Laboratory for Nonequilibrium Synthesis and Modulation of Condensed Matter, Xi’an Jiaotong University, Xi’an 710049, China

## Abstract

In this paper, the design and experimental demonstration of a snapshot linear-Stokes imaging spectropolarimeter (SLSIS) is presented. The SLSIS, which is based on division-of-focal-plane polarimetry with four parallel linear polarization channels and integral field spectroscopy with numerous slit dispersive paths, has no moving parts and provides video-rate Stokes-vector hyperspectral datacubes. It does not need any scanning in the spectral, spatial or polarization dimension and offers significant advantages of rapid reconstruction without heavy computation during post-processing. The principle and the experimental setup of the SLSIS are described in detail. The image registration, Stokes spectral reconstruction and calibration procedures are included, and the system is validated using measurements of tungsten light and a static scene. The SLSIS’s snapshot ability to resolve polarization spectral signatures is demonstrated using measurements of a dynamic scene.

Stokes imaging spectropolarimeters are versatile instruments to acquire Stokes-vector spectral signatures in a variety of scenes^1^,^2^,^3^,^4^,^5^,^6^,^7^. It combines the abilities of an imaging spectrometer and imaging polarimeter to provide a full four-dimensional (two spatial, one spectral, one polarization) datacube. The polarization dimension could be Stokes parameters (S0, S1, S2, S3) or their derivatives such as the angle of polarization and the degree of polarization^5^. The datacube can be acquired by scanning in the spatial, spectral, or polarization dimension^8^,^9^,^10^,^11^,^12^,^13^,^14^,^15^, or within a single snapshot^16^,^17^,^18^,^19^,^20^,^21^. Although time-sequential Stokes imaging spectropolarimeters with higher acquisition speed may resolve the datacube in a real time, snapshot systems are preferable candidates for simpler operation and less misregistration in dynamic process^22^.

A snapshot Stokes imaging spectropolarimeter is usually the combination of a snapshot imaging spectrometer with a snapshot imaging polarimeter. The spectral datacube could be multispectral, hyperspectral or superspectral, limited by the dispersive ability of the spectrometer. Similarly, there are diverse polarization datacubes that are subject to the polarization resolving ability of the polarimeter on the Stokes parameters. For example, polarimetric spectral intensity modulation, also known as channeled spectropolarimetry, combined with a long slit grating spectrometer enables full-Stokes instantaneous “snapshot” hyperspectral imaging with perfect channel registration^16^. However, only one-dimensional spatial information along that slit direction is acquired within a single snapshot. The computed tomography imaging channeled spectropolarimeter (CTICS) can acquire dispersed images of a two-dimensional scene with polarization information^17^. However, the spectral resolution and detection speed are limited respectively by the spectral modulation and post-processing in the CTICS. To improve spectral resolution and avoid a channeled demodulation technique, a non-scanning computed tomography imaging spectropolarimeter (NS-CTISP) was proposed for snapshot imaging and the acquisition of the full-Stokes parameters’ hyper-cubes^18^. The polarization spectral information corresponding to each pupil is reconstructed with computed tomography individually. Another snapshot computed tomography imaging full-Stokes spectropolarimeter based on polarization gratings (PGs) and quarter-wave plates (QWPs) can also addresses the limitations of the CTICS^19^. However the missing cone problem in the computed tomography technique would induce low spatial frequency spectral features. Recently, a coded aperture snapshot spectral polarization imager (CASSPI) was proposed to capture the first two Stokes parameters S0 and S1 along with their spectral-spatial signature with a large field of view^20^.

We proposed a reconfigurable telecentric imaging system that can simultaneously capture multispectral and full-Stokes parameters in a single snapshot^23^. Although the system doesn’t require computationally intensive reconstruction, its flux is relatively low due to the use of multiple filters since the spectral and polarization resolution increases with the number of filters. Recently, a novel linear-Stokes multispectral imaging sensor, which uses the division-of-focal-plane paradigm, has been proposed^24^. The spectral resolution is limited by the number of the photodetectors and the flux decreases with the increase of the photodetectors. Integral-field spectroscopy is one type of snapshot spectral imaging technique^25^,^26^,^27^,^28^,^29^,^30^, and can be combined with snapshot imaging polarimetry^30^,^31^,^32^. However, the spectral resolution is still limited by the complex channeled demodulation algorithm. In contrast, we proposed a snapshot full-Stokes imaging spectropolarimeter based on a combination of division-of-aperture polarimetry with the integral-field spectroscopy^33^. Theoretically, the reconstruction of the spectral, spatial, and polarization information does not need complex and heavy computation.

In this paper, we introduce a new snapshot linear-Stokes imaging spectropolarimeter (SLSIS), based on a combination of division-of-focal-plane polarimetry with the integral-field spectroscopy, to resolve complete linear-Stokes hyperspectral information in a single integration time without heavy computational requirements.

## General principle

**Figure 1:**
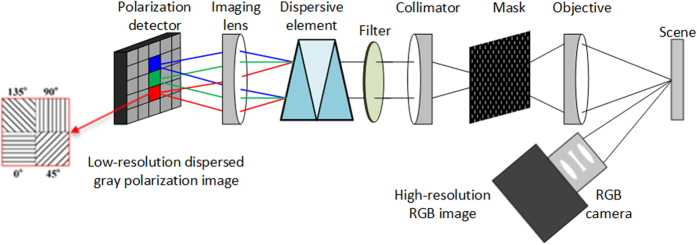
The schematic layout of the SLSIS system.

A schematic of the SLSIS is shown in Fig. 1. A scene is first imaged by an objective onto a patterned optical mask. The features on the mask could be pinholes or slits. The light from each feature is then collimated by a collimator and dispersed by a dispersive element. A bandpass filter is used to limit the detection spectral regions. An imaging lens reunites the dispersed rays on a polarization detector with a pixelated micro-polarization array. The polarization detector works as a division-of-focal-plane polarimeter and each macro pixel on the sensor consists of 2 × 2 micropolarizers. The dispersed polarization image is acquired in a single integration on the polarization detector, containing the spatial, spectral and polarization information. The dispersed polarization image can be simply remapped to obtain Stokes-vector spectral datacube or their derivative products without complex reconstruction algorithm.

In the system, the total number of the macro pixels of the detector determines the volume of the datacube. Therefore, the spectral sampling always decreases with the increase of the spatial sampling, and vice-versa. To avoid the overlap of the dispersive light, the number of features, the bandwidth of the system, the interval of the neighboring features, and the magnification of the system should be optimized carefully. Since there are only limited features on the mask, the spatial resolution is low relative to the full resolution of the polarization detector. To get a high spatial resolution for the same scene, a RGB camera is employed to assist the system in parallel as show in Fig. 1.

## System design and experimental setup

### Polarization detector

In this paper, the polarization detector is a PolarCam developed by 4D Technology Inc. The camera sensor includes 804(H) × 604(V) macro pixels and each macro pixel consists of 2 × 2 micropolarizers oriented at angles 0°, 45°, 90°, and 135°. The size of a micro pixel is 7.4 μm × 7.4 μm. This sensor provides a division-of-focal-plane polarimeter configuration and the ideal measurement matrix of each macro pixel is


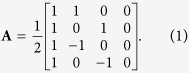


The detected intensity vector **I **= [*I*_0_, *I*_45_, *I*_90_, *I*_135_]^*T*^ at a single snapshot is


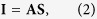


where **S** = (S0, S1, S2, S3) is the incident Stokes vector. Then the images corresponding to the incident Stokes parameters is estimated as


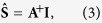


where the sign “+” indicates pseudoinverse operation. However, there will be misalignments and a relatively low extinction ratio for the micropolarizers, the actual measurement matrix of each macro pixel will deviate from the ideal matrix. Therefore, the measurement matrix of each micro pixel should be determined separately with the radiometric and polarimetric calibrations as will be discussed later.

### Dispersive element

**Figure 2:**
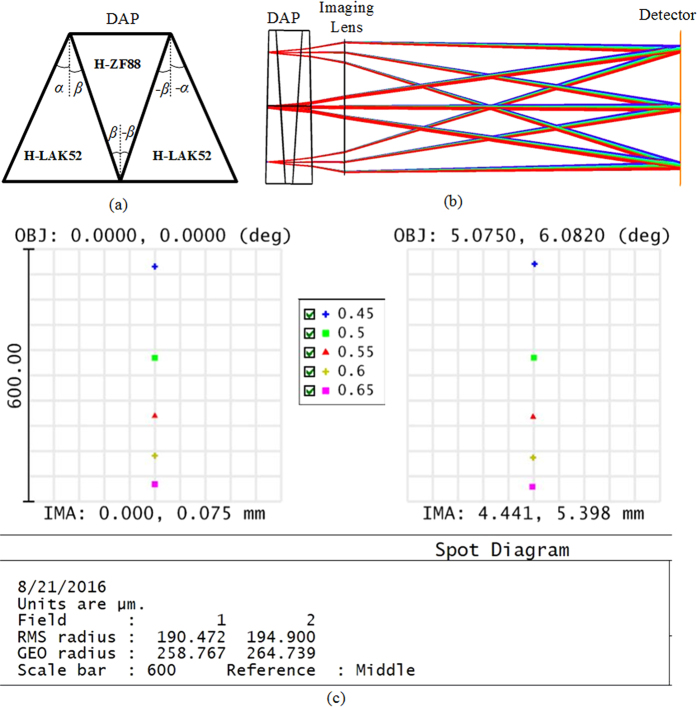
(**a**) Configuration of double Amici prism (DAP), (**b**) Ray tracing with the ZEMAX software, and (**c**) the spot diagrams at zero and maximum field of view.

The spectral and spatial distortions, known as “smile” and “keystone”, introduced by dispersive element in dispersive imaging spectrometers are usually a nuisance for the accurate reconstruction of spectra and images. Due to the specific arrangement of the micropolarizers, the distortions should be kept as small as possible. The double Amici prism (DAP) in Fig. 2(a) is a type of direct-vision dispersive element commonly used in a dispersive imaging spectrometer, the distortions can be reduced with a proper design. Since the linear distortion varies with the incident angle on the prism and the focal length of imaging lens, it is better to design the DAP with a vector ray tracing method^34^. We assume the dispersion spectral region is 450–650 nm with the central wavelength of 550 nm. The first and second elements in the DAP are respectively made of H-LAK52 and H-ZF88 from CDGM GLASS CO., LTD. The focal length of the imaging lens is *f* = 50 mm and the corresponding field of viewis approximately 5° × 6°. Optimal apex angles of the DAP are derived as *α* = 1.8° and *β* = 5.8°. Correspondingly, the dispersive length of the spectral region is *L* = 518 μm on the polarization detector. The radius of the “keystone” distortion at a maximum field of view is less than 3 μm, and the radius of the “smile” distortion is less than 6 μm. Both distortions are smaller than the size of a micro pixel. To verify our optimization results, the design parameters are imported into ZEMAX in an F#/5 system with a focal length of 50 mm. The 3-D layouts and the spot diagrams at the minimum and maximum fields of view are shown in Fig. 2(b) and (c) respectively. The dispersion lengths reported by ZEMAX agree with the optimization.

### Optical mask

**Figure 3:**
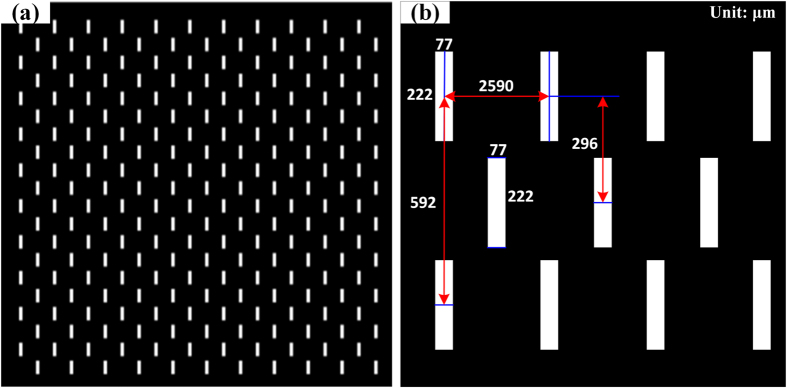
(**a**) Optical mask with rectangular slits and (**b**) the slit dimension.

In this paper, the features on the mask are slits customized from Front Range PhotoMask Co. The features are lithographically patterned as a chrome coating on a 2.5″ × 2.5″ × 0.6″ quartz substrate as shown in Fig. 3(a). To get a well-proportioned spatial sampling, there is an offset for the slits in the neighboring two rows. The size of the slit on the mask determines the spectral resolution and optical flux. The spectral resolution always decreases with the increase of the optical flux, and vice-versa. Since the macro pixel consists of the neighboring 2 × 2 micropolarizers and the total flux would be split into four portions, the optical flux should be considered first. In addition to minimizing the error of the instantaneous field of view on the 2 × 2 neighbor micro pixels^35^,^36^, the smallest size of the slits should cover the 2 × 2 micro pixels. In this paper, the size of the slit is 222 μm × 74 μm as shown in Fig. 3(b), covering 6 × 2 micro pixels. To avoid spectra overlapping between the neighboring two slits due to distortions and diffraction, the vertical interval of the two slits is 592 μm which is larger than twice the length of the slit. Since the linear dispersive length of the DAP is *L* = 518 μm, the dispersed spectra would cover 70 micro pixels (35 macro pixels) horizontally, the horizontal interval of the neighboring two slits is 2590 μm.

### Experimental setup

**Figure 4:**
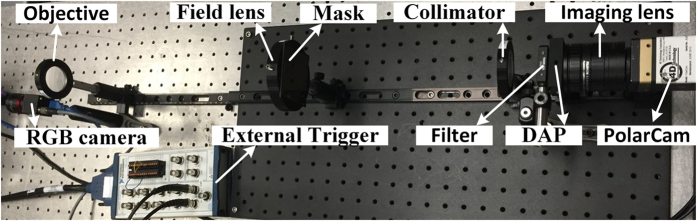
Experimental setup.

Figure 4 shows the experimental setup. The objective and collimator are achromatic doublets with a focal length of 250 mm and a diameter of 40 mm. The imaging lens has a focal length of 50 mm and a F/# of 5.6 (FUJINON CF50HA-1). A field lens with a focal length of 195 mm is used to increase the light efficiency. The spectral range of the system is limited to 450–650 nm with a bandpass filter (Semrock FF01-550/2000-25). Since the dispersed spectra *L* = 518 μm covers 35 macro pixels, the nominal spectral resolution would be 5.7 nm. The spectral resolution would decrease with the increase of wavelength due to nonlinear dispersion. An external trigger (NATIONAL INSTRUMENTS BNC-2110) is used to control the PolarCam and the RGB camera to simultaneously acquire images. The RGB camera is the Flea3 USB 3.0 digital camera (Point Grey FL3-U3-13S2C-CS) with a full resolution of 1328 (H) × 1048 (V) and the pixel size of 3.63 μm × 3.63 μm. The focal lengths of the CS-mount lens is 25 mm with a F/# of 5.6 (EDMUND Optics 67715 VIS - NIR).

### Calibration and registration

#### Calibration setup

Geometric calibration, spectral calibration, radiometric calibration and polarimetric calibration should be implemented first in order to reconstruct accurate Stokes-vector spectra from the original data. The registration between the RGB image and the polarization images is also important for locating the slit positions on the high resolution RGB image. Monochromatic light from a monochromator or polychromatic light from a light guide is transferred into a three-ports 6” integrating sphere to remove any polarization signature and produce a uniform natural light at the exit port. A step-variable metallic neutral density filter acts as a rotatable attenuator to flexibly modulate the intensity of the polychromatic light. A rotatable polarizer inserted in front of the exit port is used to produce variable linearly polarized light. A spectrometer (OCEAN Optics USB4000) is used to monitor the spectrum of the light source.

#### Geometric calibration

Although the DAP is properly designed and the system is well aligned, the dispersed polarization images deviate from the ideal ones due to the residual distortions. The images should be corrected with geometric calibration. First, we illuminate the system with a uniform monochromatic light source and the centroid of each slit is found on the images. For the dispersed images over the spectral range of 450–650 nm, it is found that the image at the wavelength of 520 nm is located at the middle position of the waveband. Therefore, the centroids of the slits will be searched from the image. Second, since the slits on the mask are regularly arranged and there is minimal distortion in the center of the image, the intervals of the centroids in the image center are computed and used to generate a series of regular reference points. Finally we match the centroids with the reference points using a polynomial fitting and produce a transformation matrix which is used to correct any distorted polarization images measured in the same circumstances.

#### Spectral calibration

**Figure 5:**
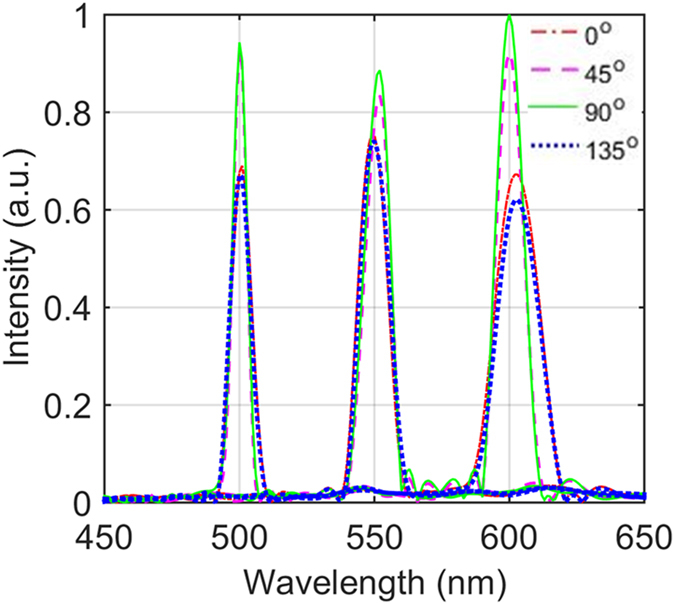
The corrected positions for the spectra of a slit at three wavelengths.

Spectral calibration is used to determine the wavelength position of the dispersed spectra of each slit. Here we use the monochromator to produce monochromatic light from 450 nm–650 nm with an interval of 10 nm. An important step is to extract the spectral image of each slit before the calibration. We first search for the coordinates of the centroids for the slits on the corrected polarization image of 520 nm. Then the spectral range is determined by horizontally extending about the centroid to 35 pixels and vertically extending the centroid to 3 pixels. Finally, the transformation coefficient between the reference wavelengths and the pixel positions of slit spectra is determined with polynomial fitting. Figure 5 shows the calibrated spectra of a slit at three wavelengths of 500 nm, 550 nm, and 600 nm. The spectra at four polarization directions (0°, 45°, 90°, and 135°) approximately overlap each other. The small deviations ( ≤ 2 nm) of the peak locations are due to the location offsets of the four micropolarizers. Since the DAP has a lower dispersive capability at longer wavebands, the deviation increases with wavelength. According to the locations of the four micropolarizers shown in Fig. 1, theoretically the wavelength positions for 0° and 135°, 90° and 45° would overlap each other. The results in Fig. 5 are consistent with these locations.

#### Radiometric calibration

**Figure 6:**
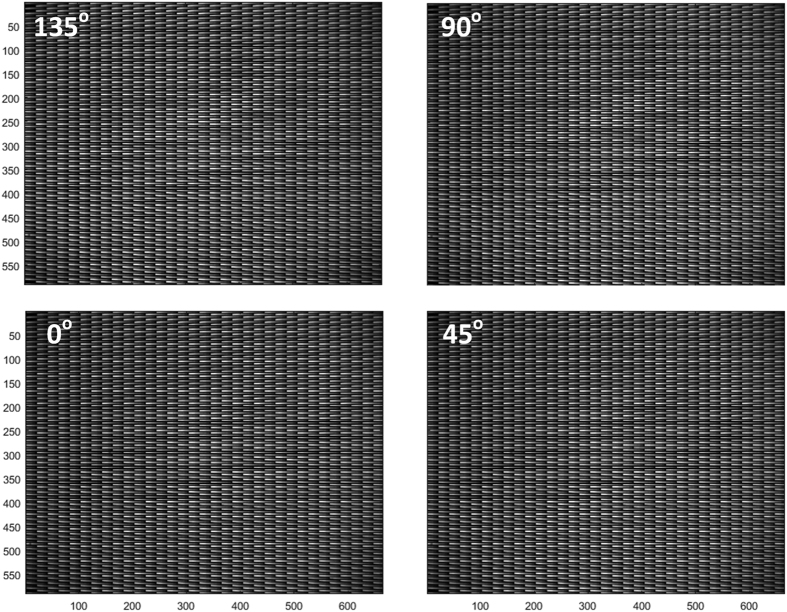
The dispersed images of the slit array for the four polarization channels (0°, 45°, 90°, and 135°) when the system is illuminated with the uniformly extended polychromatic natural light.

As shown in Fig. 5, the intensities of the spectra for different polarization channels do not agree with each other. Furthermore, the spectral responses of the slits also vary with their positions. Radiometric calibration is to let each slit has the correct spectral response and let all of slits have uniform response over the image plane. The standard polychromatic natural light is usually used for radiometric calibration. To account for the potential nonlinear response of the detector, the intensity of the reference light source is changed from high to low level with the rotatable attenuator. Correspondingly, the Ocean spectrometer and our system simultaneously record the spectra at each level of the illumination. The data of Ocean spectrometer is used as reference. We use polynomial fitting to get the transformation coefficient between the reference spectra and measured spectra for all of light levels. Figure 6 shows the uncalibrated dispersion images of the slit array when the system is illuminated with the natural polychromatic light of medial intensity level. There are about 1800 effective slits in each polarization image.

**Figure 7:**
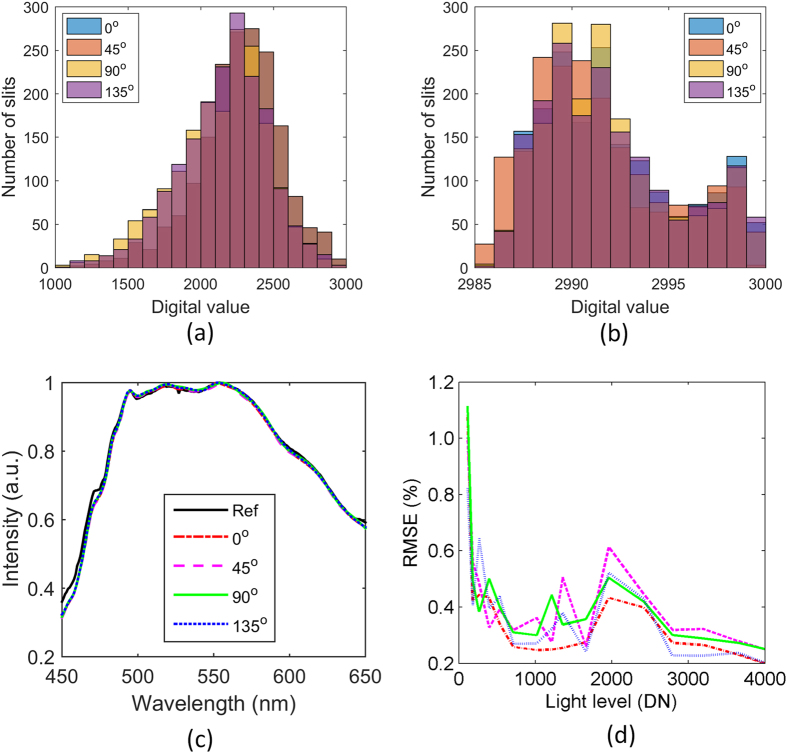
The intensity distribution of the slit array image at the wavelength of 600 nm (**a**) before calibration and (**b**) after calibration, when the system is illuminated by the natural polychromatic light with a medial light level. (**c**) The calibrated polarization spectra of a single slit. (**d**) The RMS errors of the calibrated polarization spectra of the slit under different light levels.

Figure 7(a) represents the uniformity of the slit array image at the wavelength of 600 nm. Obviously, the spectral responses of the slits are uneven in each image. However, the non-uniform distribution is well calibrated using the transformation coefficient as shown in Fig. 7(b). Figure 7(c) shows the calibrated polarization spectra from a single slit using the transformation coefficient for a media light level. The spectra of the four polarization channels approximately overlap each other. Figure 7(d) shows the root-mean-square (RMS) errors of the calibrated polarization spectra of the slit under different light levels. The abscissa represents the maximum intensities of the reference spectra under different light levels, which are normalized to the 12 bit digital values (DNs) of the CCD detector. As seen, the maximum RMS error of each polarization channel is less than 1.2%. The accuracy decreases as the light level reduces, which means the radiometric calibration with the different light levels are important for nonlinear corrections.

#### Polarization calibration

**Figure 8:**
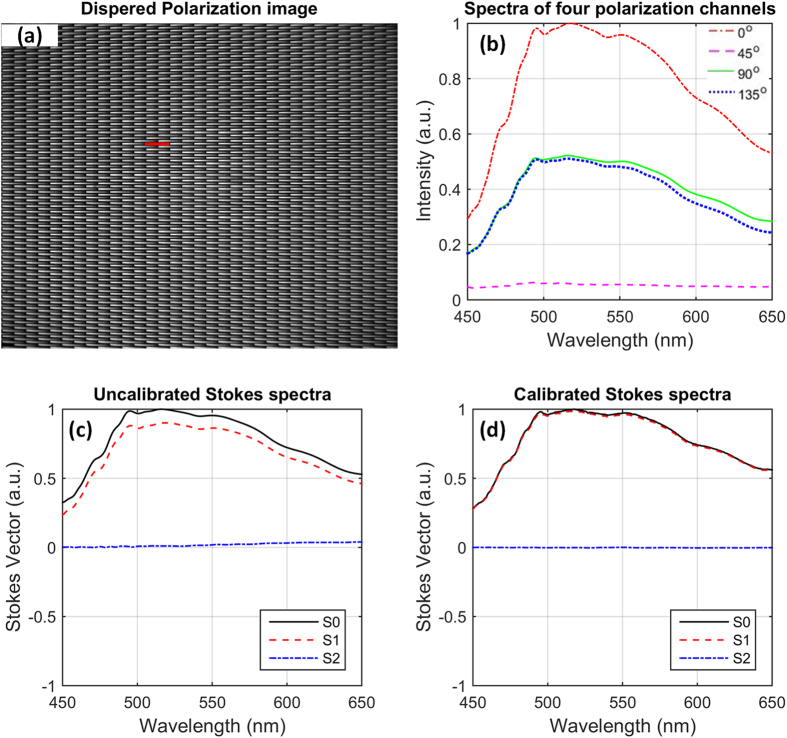
(**a**) Dispersed slit array image of the 0° polarization channel, (**b**) the spectra of the four polarization channels correspond to a slit spectrum (red bar) in (**a**), (**c**) uncalibrated Stokes spectra, and (**d**) the calibrated Stokes spectra for the illumination of a uniform 0° polarized polychromatic light.

Polarization calibration gets rid of the polarization effects of the unpolarized components and compensate the imperfect polarization components. Figure 8(b) shows the measured spectra of the four polarization channels for a dispersed slit (red bar) in Fig. 8(a) when we illuminated the system with a 0° linearly polarized polychromatic light. Theoretically, the spectra of 45° and 135° polarization channels should overlap each other and the spectra of 90° polarization channel should approach to zero. However there are small deviations due to the imperfections in the system. Therefore, the Stokes-vector spectra in Fig. 8(c) derived using Eqs (1, 2, 3) also deviate from that of the 0° linearly polarized polychromatic light. The true value of S1 should be equal to S0, and S2 should be equal to zero. The Stokes parameter S0 is already corrected during the radiometric calibration.

To account for higher order effects of the systematic errors, we directly measure the measurement matrix of the system by using a pseudo-inversion estimation with a set of well-known reference polarization states (0°, 45°, 90°, and 135°). Then according to Eq. (2), the measurement matrix is derived as


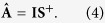


With the use of the calibrated measurement matrix in Eq. (4) instead of the ideal measurement matrix in Eq. (1), the corrected Stokes spectra are determined in Fig. 8(d). As seen, the corrected Stokes parameter S1 is now overlap with the Stokes parameter S0, and the corrected Stokes parameter S2 is equal to zero. Both the RMSEs of S1 and S2 over the whole waveband are less than 0.05%, meaning that the angle of polarization (AOP) of the calibrated spectra is zero which is equal to the 0° linearly polarized reference polychromatic light

#### Registration of the RGB camera and the spectropolarimetric camera

Since the spectropolarimetric camera only acquires the Stokes-vector hyperspectral image with a relatively low spatial resolution, the RGB camera is used to get the high spatial resolution of the same scene, as shown in Fig. 1. To locate positions on the RGB image that overlap with the slit positions, the spectropolarimetric camera and RGB camera should be aligned and registered. The procedure is as follows. The mask is first removed from the spectropolarimetric camera and an optical filter of 520 nm is inserted to get the gray image of a calibration chart. This step should be implemented before the above various calibrations to avoid any changes in the system. The geometric calibration of the RGB camera should be implemented using the same method discussed above. Then the coordinates of the gray image are transferred to the RGB image with the forward geometric transformation method. Currently, the registration accuracy is less than a pixel size of the spectropolarimetric camera.

#### Experimental verification

**Figure 9:**
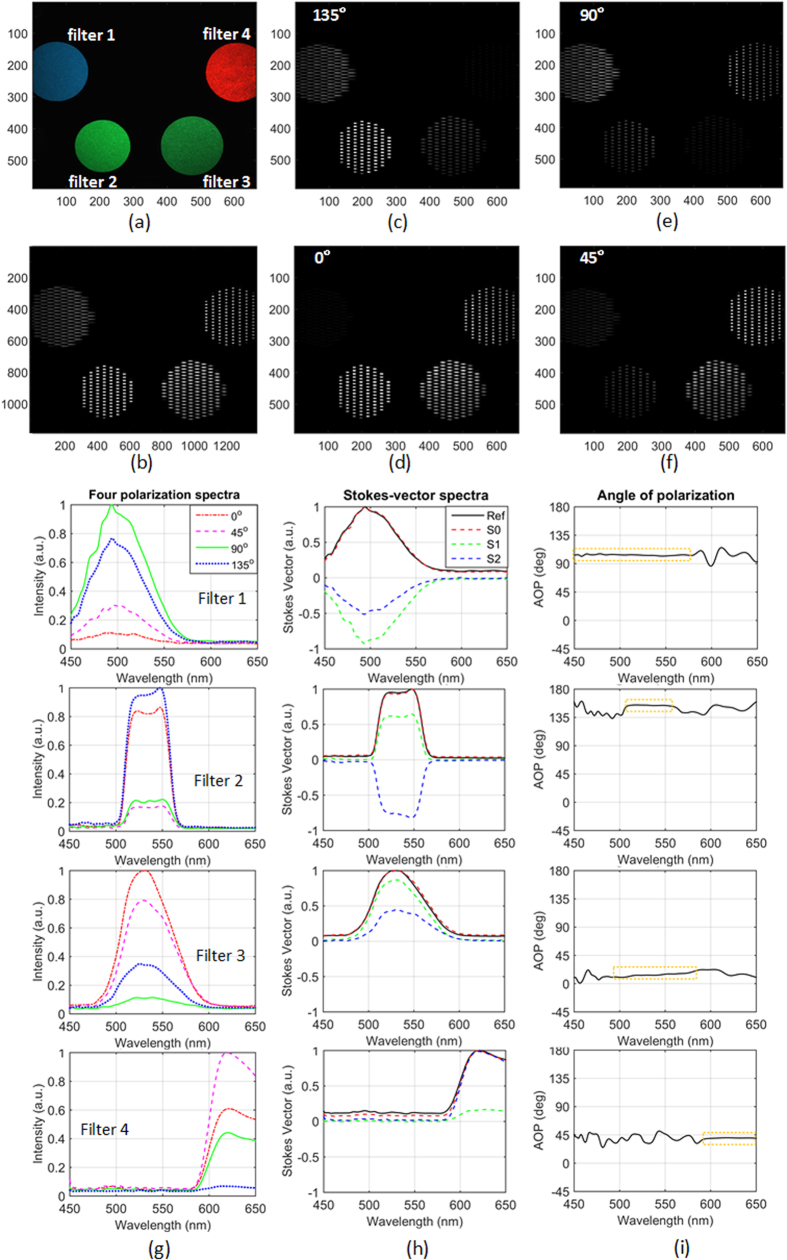
(**a**) A frame of the snapshot RGB video (Media 1). Filters 1–4 are covered with polarizers oriented at 103°, 154°, 13° and 40°. (**b**) The snapshot dispersed polarization gray image that is the hybrid of the four polarization channels, and (**c**–**f**) Four polarization channels extracted from (**b**), (**g**) is the four polarization channel spectra at different locations in (**a**), the second column (**h**) corresponds to the Stokes-vector spectra where the black solid lines indicate the reference value, and the third column (**i**) is the AOP spectra of the four slits within the four filters respectively (Media 1).

To demonstrate the snapshot spectral and polarization capabilities, we use unpolarized polychromatic light to illuminate a manually rotatable filter wheel with four filters with four linear polarizers oriented at different directions. Filters 1, 3, and 4 are Kodak Wratten color filters working at the cyan, green, and red wavebands, respectively (Edmund Optics #54-465, #53-701, #53-699), and filter 2 is a fluorescence filter with a central wavelength of 535 nm and a bandwidth of 43 nm (Edmund Optics #67-031). The four polarizers covered on the filters are visible linear polarizer film (Edmund Optics #38-497), and their orientations are random and vary with the rotation of the wheel. During the rotation of the filter wheel, the two cameras simultaneously acquire images at a speed of 10 frames per second with the control of an external trigger. Figure 7(a) shows a frame of the RGB video. For the frame in Fig. 9(a), the orientations of the polarizers on the filters 1–4 are 103°, 154°, 13° and 40° respectively. Figure 9(b) shows the dispersed image spectra from a frame of the polarization gray video that is acquired at the same time as the RGB frame. Figure 9(c–f) are the images of the four polarization channels extracted from Fig. 9(b). As shown, the filter intensities are different over the four channels and are approximately consistent with the orientations of the polarizers.

Figure 9(g) shows the four polarization spectra of four slits within the four filters, respectively. The derived Stokes-vector spectra are shown in Fig. 9(h). The spectrum of S0 agrees with the reference spectra. The AOP spectra corresponding to higher intensities (within the orange dotted rectangle) in Fig. 9(i) are stable and consistent with the orientations of the polarizers. The root-mean-square error between the AOP within the orange dotted rectangles and the ideal values is less than 5%. However, the AOP spectra corresponding to lower intensities have some vibrations, meaning the system is a little sensitive to noise^37^,^38^. The way to improve the spectra stability and accuracy for weak scenes is to increase the exposure time. Media 1 displays the slit spectra that are nearest to the clicked point in the RGB video. As shown, the AOP spectra corresponding to higher intensities vary smoothly with the rotation of the filter wheel.

## Conclusion

In conclusion, we have proposed a new snapshot linear-Stokes imaging spectropolarimeter. It integrates the advantages of division-of-focal-plane polarimetry and integral field spectroscopy to acquire the hyperspectral images of the first three Stokes parameters within a single integration time. After calibration and image registration, the reconstruction of the spatial, spectral, and polarization datacube only needs a simple remapping, compared to the systems that employ the channeled demodulation, compressive sensing or computed tomography^16^,^17^,^18^,^19^,^20^,^21^.

Since the spatial resolution is limited by the number of the slit features on the optical mask, the RGB camera is employed to assist the acquisition of the same scene with high resolution. In this paper, the Stokes-vector hyperspectral datacube of each location on the RGB image is approximately equivalent to that of the nearest slit on the grey image. The Stokes-vector hyperspectral datacube for each spatial position on the RGB image may be estimated with a bilateral interpolation filtering approach or a piece-wise Wiener estimation method^28^,^29^, although both methods are only applied to the unpolarized hyperspectral datacube.

One limitation in the current system is the polarimetric capability. Currently only the linear Stokes-vector spectra can be resolved due to the polarimetric capability of the PolarCam micropolarimeter sensor. Although full-Stokes division-of-focal-plane polarimeters were developed recently^39^,^40^, the working waveband is very narrow. Compared to the micropolarizers in the PolarCam^24^, it is challenge to fabricate wide waveband achromatic micro-retarders for the full-Stokes division-of-focal-plane polarimeters currently. Another limitation is the frame-rate of the system. For the current system, the only way to improve the frame rate is to reduce the exposure time of the PolarCam sensor, however the sensitivity would be reduced and the reconstructed spectra would be inaccurate due to the influence of noise. The vibrations of the AOP in Fig. 9 are mainly due to the low flux. With higher flux or longer integration time, the higher the accuracy and stability for the measured spectra.

## Additional Information

**How to cite this article**: Mu, T. *et al*. Snapshot linear-Stokes imaging spectropolarimeter using division-of-focal-plane polarimetry and integral field spectroscopy. *Sci. Rep.* **7**, 42115; doi: 10.1038/srep42115 (2017).

**Publisher's note:** Springer Nature remains neutral with regard to jurisdictional claims in published maps and institutional affiliations.

## Supplementary Material

Supplementary Information

Supplementary Video
