# Supplementary material for: Snapshot linear-Stokes imaging spectropolarimeter using division-of-focal-plane polarimetry and integral field spectroscopy
Source: Sci Rep. 2017 Feb 13;7:42115. doi: 10.1038/srep42115 (PMC5304160; doi:10.1038/srep42115)
Supplement: Supplementary Information [file srep42115-s1.pdf]

Snapshot linear-Stokes imaging spectropolarimeter using division-of-focal-plane polarimetry and integral field spectroscopy

Tingkui Mu,[1,2,3] Shaun Pacheco,[1] Zeyu Chen,[2] Chunmin Zhang,[2] and Rongguang Liang[1,*]

[1]College of Optical Sciences, University of Arizona, Tucson, Arizona 85721, USA
[2]Institute of Space Optics, School of Science, MOE Key Laboratory for Nonequilibrium Synthesis and Modulation of Condensed Matter, Xi'an Jiaotong University, Xi'an 710049, China
[3]tkmu@mail.xjtu.edu.cn
[*]rliang@optics.arizona.edu

Media 1: Demonstration of the snapshot spectral and polarization capabilities with a rotatable filter wheel with four filters with four linear polarizers oriented at different directions
